# Physical Frailty Versus the MECKI Score in Risk Stratification of Patients with Advanced Heart Failure: Simpler Measure, Similar Insights?

**DOI:** 10.3390/jcm15020513

**Published:** 2026-01-08

**Authors:** Francesco Curcio, Rosaria Chiappetti, Cristiano Amarelli, Irene Mattucci, Allegra Di Somma, Francesca Maria Stagnaro, Federica Trotta, Gennaro Alessio, Seyedali Ghazihosseini, Ciro Abete, Ciro Maiello, Pasquale Abete, Francesco Cacciatore

**Affiliations:** 1 Department of Translational Medical Sciences, University of Naples “Federico II”, 80131 Naples, Italy; 2 Department of Cardiac Surgery and Transplants, Monaldi Hospital—Azienda dei Colli, 80131 Naples, Italy

**Keywords:** frailty, heart failure, MECKI, cardiopulmonary exercise testing

## Abstract

**Background/Objectives:** Frailty, a syndrome characterized by diminished physiological reserves and increased vulnerability to stressors, is a strong predictor of adverse outcomes in heart failure. The MECKI (Metabolic Exercise Cardiac Kidney Index) score, derived from cardiopulmonary exercise testing and renal function parameters, has demonstrated prognostic value in HF patients. This study aimed to evaluate the prognostic value of physical frailty on mortality in patients with advanced heart failure and to compare it directly with the MECKI score. **Methods:** A total of 104 patients with advanced HF receiving optimized guideline-directed medical therapy were prospectively enrolled. At baseline, all patients underwent clinical, echocardiographic, and laboratory assessment and CPET for MECKI score calculation. Physical frailty was assessed using a modified Fried phenotype tailored for HF. The composite endpoint comprised all-cause mortality, urgent heart transplantation, or LVAD implantation. **Results:** Over a mean follow-up of 30.0 ± 15.3 months, there were 25 deaths, 5 urgent heart transplants, and 1 LVAD implantation. Patients who experienced the composite outcome had significantly worse NYHA class, higher NT-proBNP, lower VO_2_max, higher VE/VCO_2_ slope, higher frailty, and higher MECKI score (all *p* < 0.001). Frailty was significantly correlated with all MECKI score components, as demonstrated by Spearman’s rank correlation analysis. Both frailty (HR = 1.89; 95% CI 1.22–2.93; *p* = 0.005) and MECKI score (HR = 1.04; 95% CI 1.00–1.08; *p* = 0.037) independently predicted outcomes. ROC analysis showed high and comparable discriminative performance (AUC = 0.86 for frailty; AUC = 0.88 for MECKI). **Conclusions:** Physical frailty and MECKI scores independently predict mortality and adverse events in advanced HF. Physical frailty, despite its simplicity and low cost, provides prognostic insight comparable to the MECKI score and may represent a practical alternative when CPET is unavailable.

## 1. Introduction

Frailty is increasingly recognized as a major determinant in the management of patients with heart failure (HF) [1]. Both HF and frailty are highly prevalent conditions, particularly with advancing age, and are associated with adverse prognostic implications [2]. It is estimated that approximately 50% of patients with HF are frail, although prevalence estimates vary widely depending on patient characteristics and the frailty assessment tools used [3]. Two main frailty paradigms have been described: the physical (or phenotypic) model (PF) [4], which focuses on measurable physical decline, and the multidimensional model (MF) [5], which also incorporates cognitive, psychological, and social domains. Despite ongoing debate regarding the optimal definition and assessment of frailty, its presence is strongly associated with worse outcomes in HF, including an approximately 50% higher risk of hospitalization and mortality compared with non-frail individuals [6]. Frailty has also been linked to more severe symptoms, such as dyspnea, and poorer quality of life in patients with HF [7]. In advanced heart failure (AHF), frailty is particularly prevalent and significantly influences prognosis in patients undergoing heart transplantation (HT) [8] or left ventricular assist device (LVAD) implantation [9].

Alongside frailty assessment, the Metabolic Exercise Cardiac Kidney Index (MECKI) score has emerged as a prognostic tool in HF [10]. The MECKI score integrates six variables: hemoglobin, serum sodium, renal function, left ventricular ejection fraction, percentage of predicted peak oxygen consumption (VO_2_max), and the minute ventilation–carbon dioxide production slope (VE/VCO_2_) [11]. This multiparametric score provides an integrated estimate of cardiovascular mortality risk and the need for urgent HT. Recently, the MECKI score has been validated in an international multicenter cohort [12]. However, its application is limited by the requirement for cardiopulmonary exercise testing (CPET), which is not feasible in a substantial proportion of patients with AHF.

Although both physical frailty and the MECKI score have demonstrated prognostic relevance in AHF, direct comparisons of their respective prognostic performance remain limited. Therefore, the present study aimed to assess and compare the prognostic value of PF and the MECKI score in patients with AHF. While the MECKI score relies on advanced cardiopulmonary and renal parameters to estimate risk, PF represents a simpler and more accessible assessment that may capture additional dimensions of patient vulnerability not fully reflected by complex physiological scoring systems. By clarifying the relative and complementary prognostic value of these two tools, this study seeks to improve risk stratification and support more individualized clinical decision-making in this high-risk population.

## 2. Materials and Methods

### 2.1. Study Population

One hundred thirty-four patients with AHF were consecutively enrolled from November 2018 to April 2021 and were prospectively followed at the Department of Cardiovascular Emergencies, Clinical Cardiorespiratory Medicine, and Geriatrics of the “Federico II” University Hospital in Naples and at the Heart Transplant Centre of the “Monaldi Hospital”. Twenty patients were unable to perform CPET and were therefore excluded, resulting in a sample of 114 patients. Baseline clinical characteristics of patients excluded because of an inability to perform CPET were compared with those of the study cohort and are reported in Supplementary Table S1.

Within the overall population evaluated at the Heart Transplant Center of the “Monaldi” Hospital in Naples, 43 patients were listed for heart transplantation, 15 were under evaluation for listing, and 56 were excluded from the waiting list due to comorbidities or age limits. All patients were evaluated for optimization of medical therapy according to current guidelines at the time of study inclusion.

Patients underwent laboratory testing, including measurement of N-terminal pro–B-type natriuretic peptide (NT-proBNP). Additionally, each patient underwent a comprehensive clinical evaluation, including cardiological assessment with transthoracic echocardiography and interrogation of any implanted cardioverter-defibrillator (ICD), as well as functional evaluation with CPET. The MECKI score was calculated at the time of study inclusion.

Clinical outcomes, including mortality, urgent heart transplantation, and LVAD implantation, were assessed through December 2024. During follow-up, 4 patients were lost to follow-up, and 11 underwent heart transplantation; among these, 6 elective procedures were excluded from the outcome analysis. The final study population therefore comprised 104 patients.

### 2.2. Frailty Evaluation

Frailty was assessed using an adapted version of Fried’s frailty phenotype, specifically tailored for patients with AHF awaiting HT [8]. Frailty categories were defined as robust, pre-frail, or frail according to established criteria from the original frailty phenotype literature [4]. Frailty was defined as the presence of three or more criteria among five specific measures: exhaustion, weak grip strength, reduced mobility, poor appetite, and physical inactivity. Patients meeting fewer than three frailty criteria were categorized as either robust (0 criteria) or pre-frail (1–2 criteria).

In contrast to Fried’s original approach, the assessment of appetite was prioritized over unintentional weight loss due to challenges in accurately discerning the latter in the setting of heart failure, given the impact of fluid retention. Appetite evaluation involved asking participants about their dietary intake over the preceding three months, with diminished intake indicating poor appetite. Exhaustion was determined through questions regarding feelings of fatigue and difficulty initiating tasks within the past week. Grip strength was measured using a dynamometer, with weakness defined as grip strength below two standard deviations of sex- and age-adjusted normative values. Walking speed was assessed using a 5 m walk test, with slowness defined as taking six seconds or more to complete the distance.

This adapted PF index ranged from 0 to 5, with higher scores indicating greater levels of frailty. All assessments were conducted by trained clinicians who were blinded to CPET data.

### 2.3. MECKI Score

The MECKI score was calculated considering hemoglobin, sodium, renal function (estimated Glomerular Filtration Rate (eGFR) calculated using the MDRD formula), left ventricular ejection fraction (LVEF), peak oxygen consumption (VO_2_max—percentage predicted), and ventilatory efficiency during exercise (VE/VCO_2_). It was computed as follows =10.3464 + (−0.0262 × ppVO_2_) + (0.0472 × VE/VCO_2_ slope) + (−0.1086 × Hb) + (−0.0615 × Na) + (−0.0699 × LVFE) + (−0.0136 × MDRD) [11].

For illustrative and descriptive purposes, MECKI score values were stratified into tertiles based on their distribution in the study population.

### 2.4. Statistical Analysis

Baseline characteristics of the study sample are expressed as mean ± standard deviation for continuous variables. Comparisons between two groups were performed using Student’s *t*-test or the Mann–Whitney U test for continuous variables and the χ^2^ test for categorical variables, as appropriate. When comparing clinical and functional characteristics across the three frailty categories (robust, pre-frail, and frail), one-way ANOVA was applied, followed by Bonferroni-adjusted post hoc pairwise comparisons when significant.

Correlations between the PF index and individual MECKI score components were evaluated using Spearman’s rank correlation coefficient (ρ), given the ordinal nature of the frailty score.

Follow-up was conducted for all enrolled patients, with mortality and urgent heart transplantation or LVAD implantation considered as a composite outcome. Cox proportional hazards regression analysis was performed to assess the independent prognostic value of physical frailty and the MECKI score with respect to the composite outcome. Multivariable analyses were adjusted for age, sex, and NT-proBNP, selected a priori based on clinical relevance. Internal validation was performed using bootstrap resampling to assess the stability of regression coefficients. As a sensitivity analysis, Cox regression was also performed using mortality as a standalone endpoint, censoring patients at the time of urgent heart transplantation or LVAD implantation.

Receiver operating characteristic (ROC) curves were constructed to evaluate the area under the curve (AUC) for the physical frailty index and the MECKI score in relation to the composite outcome. The discriminative performance of physical frailty and the MECKI score was compared using DeLong’s test for correlated ROC curves. Statistical analyses were performed using SPSS software (version 27.0; IBM Corp., Armonk, NY, USA).

## 3. Results

### 3.1. Patient Characteristics and Clinical Outcomes

The study included 104 patients with AHF, with a mean age of 58.4 ± 12.0 years; 84.6% were male. The mean LVEF was 26.5 ± 6.6%, and the mean NT-proBNP level was 2757.1 ± 2337.5 ng/L. Functional capacity was markedly reduced, with a mean VO_2_max of 12.1 ± 3.7 mL/kg/min and a mean VE/VCO_2_ slope of 37.6 ± 8.6. The mean PF index was 2.2 ± 1.6, and the mean MECKI score was 19.6 ± 12.9.

Patients excluded due to inability to perform CPET exhibited a distinct clinical profile compared with the study population. As reported in Supplementary Table S1, these patients were older and had worse renal function, lower hemoglobin and sodium levels, and a higher degree of PF, whereas left ventricular systolic function and right ventricular parameters were largely comparable.

During a mean follow-up of 30.0 ± 15.3 months, 25 patients (24.0%) died, 5 underwent urgent HT, and 1 received an LVAD, resulting in a total of 31 adverse events (29.8%). The composite endpoint included mortality, urgent heart transplantation, or LVAD implantation.

### 3.2. Clinical, Functional, and Frailty Profiles in Relation to Outcomes

Patients who experienced the composite endpoint showed significantly worse clinical, functional, and frailty profiles (Table 1). They had a higher New York Heart Association (NYHA) functional class (3.1 ± 0.4 vs. 2.8 ± 0.6; *p* = 0.008), lower VO_2_max (9.8 ± 2.4 vs. 13.1 ± 3.7 mL/kg/min; *p* < 0.001), and higher NT-proBNP levels (4691.9 ± 2816.3 vs. 1935.5 ± 1484.5 ng/L; *p* < 0.001). Both the PF index (3.7 ± 1.1 vs. 1.6 ± 1.4; *p* < 0.001) and the MECKI score (32.0 ± 11.3 vs. 14.3 ± 9.4; *p* < 0.001) were significantly higher in patients with adverse outcomes.

When stratified by frailty category, a progressive deterioration in clinical, functional, and echocardiographic parameters was observed with increasing frailty. However, frailty was not associated with major differences in medical therapy, except for the use of furosemide (94.1% in frail, 93.3% in pre-frail, and 65.2% in robust patients; *p* for trend = 0.002) and amiodarone (47.1%, 40.0%, and 13.0%, respectively; *p* for trend = 0.019) (Table 2).

### 3.3. Prognostic Value of Frailty and MECKI Score

Frailty was significantly correlated with all components of the MECKI score. Higher frailty scores were associated with lower hemoglobin, sodium, eGFR, LVEF, and VO_2_max, and with a higher VE/VCO_2_ slope (all *p* < 0.01), indicating a consistent worsening of cardiac, renal, and functional status with increasing frailty levels (Table 3).

In the Cox regression model (Table 4), physical frailty emerged as an independent predictor of mortality (hazard ratio [HR] 1.84; 95% confidence interval [CI] 1.25–2.72; *p* = 0.002), independent of age, sex, NT-proBNP, and the MECKI score. The MECKI score also independently predicted mortality (HR 1.04; 95% CI 1.00–1.08; *p* = 0.014).

Bootstrap resampling confirmed the stability of the regression coefficients and the independent association of both PF and the MECKI score with the outcome (Supplementary Table S2). In sensitivity analyses restricted to all-cause mortality, both PF and the MECKI score remained significantly associated with the outcome, with effect estimates consistent with those observed for the composite endpoint (Supplementary Table S3).

**Figure 1:**
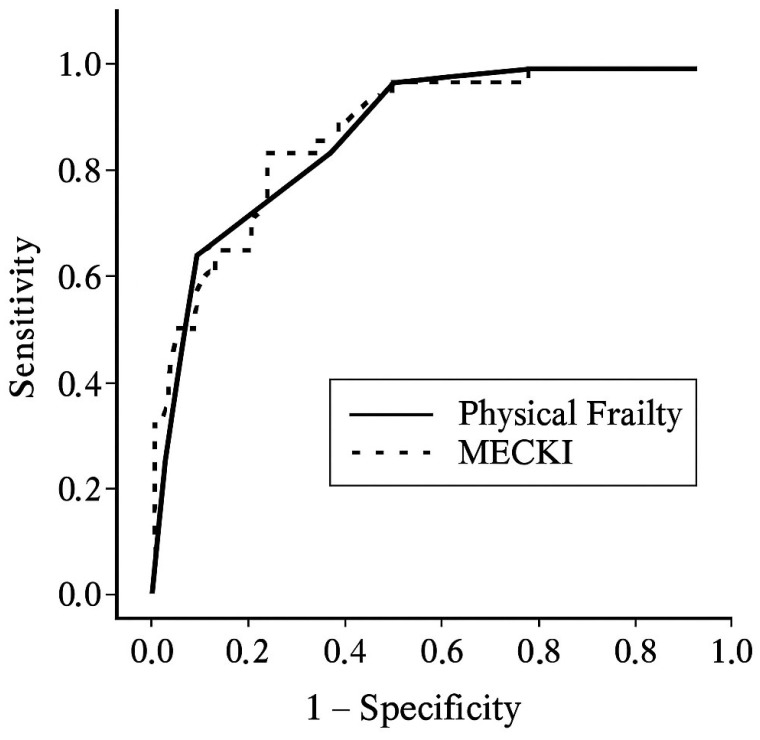
Receiver operating characteristic (ROC) curves for predicting adverse outcomes using the PF Index (solid line) and the MECKI score (dashed line).

ROC curve analysis (Figure 1) showed excellent discrimination for both the PF index (AUC = 0.866) and the MECKI score (AUC = 0.883). Formal comparison using DeLong’s test for correlated ROC curves revealed no statistically significant difference between the two AUCs (*p* = 0.721).

**Figure 2:**
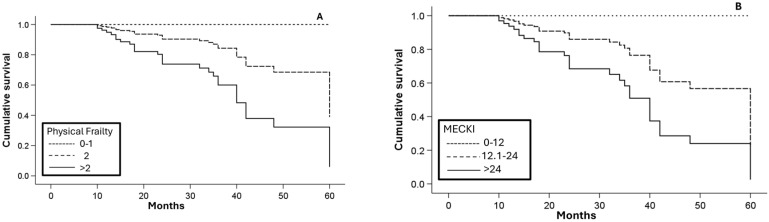
Kaplan–Meier survival analysis according to Physical Frailty (**A**) and MECKI score (**B**). Patients were stratified according to frailty categories (robust 0–1, pre-frail 2, frail > 2) and MECKI score tertiles based on the study population distribution.

Kaplan–Meier analysis (Figure 2) confirmed progressively lower event-free survival across increasing frailty categories and MECKI score tertiles.

## 4. Discussion

The results of this study demonstrate that both frailty and the MECKI score are independent predictors of adverse cardiovascular outcomes, including mortality, in patients with AHF. These associations remained significant even after adjustment for established prognostic markers, underscoring the incremental prognostic value of incorporating frailty assessment and multiparametric risk stratification into routine clinical evaluation. This evidence highlights the need for a comprehensive approach that considers not only traditional cardiovascular risk factors but also broader indicators of patient vulnerability and physiological reserve.

### 4.1. Frailty and Heart Failure

Frailty assessment in patients with HF is increasingly recognized as a valuable component of clinical practice, given the strong association between frailty and adverse outcomes in this population. Over the past decade, a growing body of evidence has underscored the prognostic and therapeutic relevance of frailty in HF, prompting guideline recommendations to routinely assess frailty in these patients [13].

The FRAGILE-HF study, a multicenter prospective cohort study, investigated the prevalence and prognostic impact of both physical and social frailty in older adults hospitalized for HF [14]. It demonstrated that frail patients had more than double the risk of one-year mortality and nearly twice the risk of one-year all-cause readmission. However, the inclusion of only hospitalized patients may have contributed to the notably high prevalence of frailty observed in this cohort (76%).

A meta-analysis by Yang et al. comparing frail and non-frail HF patients confirmed these findings, showing that frailty was significantly associated with increased risks of all-cause mortality (HR 1.54, 95% CI 1.34–1.75) and all-cause hospitalization (HR 1.56, 95% CI 1.36–1.78), with follow-up durations of 1.8 and 1.1 years, respectively. Notably, studies employing the Fried frailty phenotype reported an even stronger association with mortality (HR 1.80, 95% CI 1.41–2.28) [6].

More recently, an international multidisciplinary expert panel from the Heart Failure Association, the Korean Heart Failure Society, and the Chinese Heart Failure Society developed the Heart Failure Frailty Score (HFFS), a multidimensional frailty assessment tool created using the Delphi consensus method [15]. However, this tool has yet to be validated, and its prognostic utility in routine clinical practice remains to be established.

Despite growing consensus on the importance of frailty assessment, debate persists regarding the optimal approach—MF versus PF—in the context of HF. MF incorporates clinical, cognitive, and psychosocial domains, offering a more holistic assessment that may enhance prognostic stratification [16]. In contrast, PF remains a pragmatic and clinically relevant tool due to its simplicity, feasibility, and close alignment with the pathophysiology of HF [17].

Unlike MF, which requires comprehensive and time-intensive evaluations, PF can be assessed efficiently during routine clinical visits. This facilitates timely identification of frail and pre-frail individuals without the need for extensive resources. PF categorizes patients into well-defined phenotypes—robust, pre-frail, and frail—enabling actionable decision-making in clinical settings. Importantly, the key components of PF—fatigue, weakness, slowness, anorexia, and low physical activity—closely mirror common HF symptoms. This overlap reflects shared pathophysiological mechanisms, including systemic inflammation, hormonal dysregulation (e.g., reduced anabolic hormone levels), mitochondrial dysfunction, and impaired skeletal muscle perfusion [18]. These mechanisms contribute to sarcopenia and reduced exercise capacity, reinforcing the role of PF as a surrogate marker of HF severity and prognosis [19].

A key advantage of PF assessment in HF is its ease of implementation and greater discriminative power compared with traditional functional classifications such as the NYHA class. While the NYHA class remains widely used to assess symptom burden and functional status, it is inherently subjective, prone to inter-observer variability, and limited in capturing the progressive functional decline typical of patients with HF. In contrast, PF relies on objective and standardized metrics—gait speed, grip strength and physical activity level—which directly reflect core determinants of physical performance in HF. Although symptom overlaps exist between PF and HF, frailty has been shown to independently predict adverse outcomes even after adjustment for NYHA class and other confounding variables [20]. This supports the utility of the frailty phenotype in identifying patients experiencing accelerated biological aging beyond the effects of HF alone.

Moreover, several studies have demonstrated that PF is a stronger predictor of adverse outcomes—such as hospitalization, disability, and mortality—than NYHA class [21]. PF may capture early signs of functional decline not readily apparent through symptom-based classifications. For example, a patient classified as NYHA class II may exhibit pronounced frailty features, such as reduced muscle strength or mobility limitations, indicating a higher risk profile than suggested by NYHA class alone. Additionally, PF may serve as an early warning marker, guiding timely interventions such as tailored exercise programs or nutritional support, which may improve frailty status as well as HF outcomes [22].

Nonetheless, PF assessment has limitations. It focuses exclusively on the physical dimension of frailty, may be less sensitive to longitudinal changes, and may fail to capture the complexity of vulnerability in patients with multimorbidity. In contrast, MF offers a more nuanced understanding of patient risk but may be less feasible for routine clinical use due to its complexity and resource requirements [23].

### 4.2. Interplay Between the MECKI Score and Physical Frailty in Heart Failure

The MECKI score is a well-established prognostic tool in HF, combining CPET parameters with clinical and laboratory data to estimate risk. Despite its robust predictive value, its implementation in routine clinical practice is often limited by technical and logistical constraints [24]. Cardiopulmonary exercise testing requires specialized equipment, trained personnel, and active patient participation—resources that may not be readily available in many care settings, particularly for older, frail, or functionally impaired patients [25].

In contrast, frailty assessment—particularly the evaluation of PF—emerges as a highly accessible, cost-effective, and broadly applicable alternative. PF can be assessed by healthcare providers using simple, validated tools that require minimal training and no advanced technology. Parameters such as gait speed, handgrip strength, and physical activity level can be easily evaluated during a routine outpatient visit, making physical frailty an attractive screening and prognostic tool in both primary care and cardiology settings.

In the present study, we observed a strong correlation between PF and the components of the MECKI score, suggesting that PF may serve as a simpler and readily available surrogate with comparable prognostic performance. Furthermore, PF assessment is feasible across the full spectrum of HF patients, including those unable to undergo CPET because of advanced age, comorbidities, physical limitations, or advanced disease. This inclusivity represents a key advantage in real-world clinical practice, where a substantial proportion of patients with heart failure are elderly, multimorbid, and frequently excluded from advanced functional testing. Although PF and the MECKI score were correlated, this association likely reflects overlapping pathophysiological domains rather than statistical redundancy. Importantly, both variables remained independently associated with outcomes in multivariable Cox regression analysis, with coefficient stability supported by bootstrap internal validation, arguing against clinically relevant collinearity. Furthermore, in this context, the inverse association observed between age and the composite outcome warrants cautious interpretation, as it likely reflects eligibility for advanced therapies—such as urgent HT or LVAD implantation—rather than a protective biological effect of age.

Importantly, PF directly reflects functional domains such as skeletal muscle strength, physical endurance, and activity tolerance—areas profoundly affected by heart failure pathophysiology, including reduced cardiac output, systemic inflammation, neurohormonal activation, and metabolic imbalance [26]. PF therefore represents a sensitive and pathophysiologically meaningful marker of disease severity.

Given its simplicity and minimal resource requirements, PF assessment can be seamlessly integrated into routine outpatient care. It provides objective and clinically relevant information that complements traditional tools such as the NYHA classification, aiding in treatment personalization, rehabilitation planning, and patient monitoring.

While the MECKI score offers detailed insight into cardiorespiratory performance and systemic involvement, including renal function and congestion, PF captures a broader view of patient vulnerability, encompassing musculoskeletal, functional, and systemic impairments closely linked to adverse outcomes in HF. These two approaches should therefore be viewed as complementary rather than mutually exclusive. In particular, when CPET is not feasible, PF remains a valuable and practical alternative capable of providing meaningful prognostic information.

### 4.3. Limitations of the Study

This study has several limitations. First, the direct comparison between physical frailty and the MECKI score relied on CPET, which led to the exclusion of patients in whom this evaluation was not feasible. These patients represented a clinically distinct subgroup characterized by older age, greater symptom burden, worse renal and metabolic profiles, and a markedly higher degree of PF, despite similar indices of cardiac systolic function. As such, the study population may represent a relatively fitter subset of patients with advanced heart failure. Importantly, this selection bias is likely to attenuate, rather than inflate, the observed prognostic value of frailty. However, because outcome data were not systematically collected for the excluded group, no definitive inference can be made. Future studies specifically focusing on CPET-ineligible patients are therefore warranted.

Second, frailty was assessed using an adapted version of the Fried phenotype, in which unintentional weight loss was replaced by poor appetite to minimize misclassification related to fluid retention and congestion in advanced heart failure. Although this approach is clinically motivated and has been previously applied in the context of heart transplantation evaluation, it may limit direct comparability with studies strictly adhering to the original Fried criteria. Accordingly, our findings should be interpreted within the context of this adapted frailty definition, and further work is needed to validate and standardize frailty assessment specifically in advanced heart failure populations.

Third, the number of outcome events was relatively limited. Although internal validation using bootstrap resampling supported the stability of the multivariable model and the robustness of the observed associations, effect estimates should be interpreted with caution and confirmed in larger cohorts.

Finally, urgent heart transplantation and LVAD implantation were included in the composite endpoint and may be influenced by clinical decision-making and resource availability. However, sensitivity analyses restricted to all-cause mortality yielded consistent results, supporting the robustness of the main findings.

## 5. Conclusions

PF provides an objective, sensitive, and clinically meaningful evaluation of functional status in patients with AHF. By targeting performance-limiting factors, it enables accurate risk stratification and supports individualized care planning in this vulnerable population.

In this study, we demonstrated that frailty assessment shows prognostic accuracy comparable to that of the MECKI score. While the MECKI score remains a robust and validated tool for risk stratification in HF, frailty assessment represents a more pragmatic, resource-efficient, and broadly applicable alternative. Its simplicity and applicability across all stages and phenotypes of HF underscore its role as a key component of patient-centered care, particularly in settings where access to cardiopulmonary exercise testing is limited.

## Figures and Tables

**Table 1:** Comparison of clinical characteristics between event-free survivors and patients with adverse outcomes.

	All (# 104)	Event Free Survivors (# 73)	Adverse Event Group * (# 31)	*p*
Age (years, mean ± SD)	58.4 ± 12.0	57.4 ± 12.8	60.8 ± 9.6	0.186
Male (%)	84.6	86.3	80.6	0.465
NYHA class (mean ± SD)	2.9 ± 0.6	2.8 ± 0.6	3.1 ± 0.4	<0.008
LVEF (%, mean ± SD)	26.5 ± 6.6	27.8 ± 6.4	23.5 ± 6.1	<0.002
TAPSE (mm, mean ± SD)	16.9 ± 4.0	18.0 ± 3.8	14.5 ± 3.5	<0.001
PASP (mmHg, mean ± SD)	43.8 ± 14.6	39.2 ± 13.2	54.3 ± 12.4	<0.001
IVCd (mm, mean ± SD)	20.0 ± 4.8	19.7 ± 5.3	20.9 ± 3.3	0.239
VO_2_max (mL/kg/min, mean ± SD)	12.1 ± 3.7	13.1 ± 3.7	9.8 ± 2.4	<0.001
VE/VCO_2_ (slope, mean ± SD)	37.6 ± 8.6	35.7 ± 7.7	42.0 ± 9.1	<0.001
NT-proBNP (ng/L, mean ± SD)	2757.1 ± 2337.5	1935.5 ± 1484.5	4691.9 ± 2816.3	<0.001
Hemoglobin (g/dL, mean ± SD)	12.9 ± 1.7	13.2 ± 1.7	12.2 ± 1.7	0.005
Sodium (mEq/L, mean ± SD)	138.1 ± 3.6	138.9 ± 3.3	136.2 ± 3.5	<0.001
eGFR (mL/min/1.73 m^2^, mean ± SD)	61.4 ± 24.0	67.6 ± 24.4	46.6 ± 15.3	<0.001
Frailty	2.2 ± 1.6	1.6 ± 1.4	3.7 ± 1.1	<0.001
MECKI (mean ± SD)	19.6 ± 12.9	14.3 ± 9.4	32.0 ± 11.3	<0.001
MitraClip (%)	6.7	8.2	3.2	0.325
ICD (%)	84.6	78.1	100	0.005
CRT (%)	37.5	41.1	29.0	0.245
Sacubitril/valsartan (%)	76.9	78.1	74.2	0.667
Furosemide (%)	87.5	83.6	96.8	0.062
Amiodarone (%)	37.5	24.7	67.7	0.001
Metolazone (%)	4.8	4.1	6.5	0.610
Ivabradine (%)	5.8	2.7	12.9	0.043
SGLT2 inhibitors (%)	52.9	61.6	32.3	0.006
MRAs (%)	76.5	78.1	72.4	0.543
Digoxin (%)	10.6	8.2	16.1	0.230
Beta-blockers (%)	90.4	93.2	83.9	0.142

* Composite outcome of death, urgent heart transplant, and left ventricular assisting device implantation. NYHA = New York Heart Association, LVEF = left ventricular ejection fraction, TAPSE = Tricuspid Annular Plane Systolic Excursion, PASP = Pulmonary Arterial Systolic Pressure, IVCd = Inferior Vena Cava diameter, VO_2_max = maximal oxygen consumption, VE/VCO_2_ = minute ventilation/carbon dioxide production, NT-proBNP = N-terminal fragment of pro brain natriuretic peptide, eGFR = estimated Glomerular Filtration Rate, MECKI = Metabolic Exercise test data combined with Cardiac And Kidney Indexes, ICD = Implantable Cardioverter Defibrillator, CRT = Cardiac Resynchronization Therapy, SGLT2 = Sodium-Glucose Transport Protein 2, MRAs = Mineralocorticoid receptor antagonist. CRT and ICD categories are not mutually exclusive. *p*-values are provided for descriptive purposes only and were not adjusted for multiple comparisons.

**Table 2:** Clinical characteristics of the study cohort according to frailty level.

	Robust (# 23)	Pre-Frail (# 30)	Frail (# 51)	*p*
Age (years, mean ± SD)	47.7 ± 14.4	58.4 ± 7.1	63.2 ± 9.9	<0.001
Male (%)	73.9	93.3	84.3	0.435
NYHA class (mean ± SD)	2.4 ± 0.7	3.0 ± 0.4	3.0 ± 0.5	<0.001
LVEF (%, mean ± SD)	31.6 ± 6.0	25.9 ± 5.2	24.6 ± 6.5	<0.001
TAPSE (mm, mean ± SD)	19.3 ± 4.1	16.9 ± 3.3	15.9 ± 3.9	0.003
PASP (mmHg, mean ± SD)	34.0 ± 9.6	42.6 ± 13.1	48.9 ± 15.1	<0.001
IVCd (mm, mean ± SD)	18.7 ± 4.4	18.9 ± 4.4	21.3 ± 5.0	0.029
VO_2_max (mL/kg/min, mean ± SD)	15.6 ± 3.4	12.5 ± 2.9	10.4 ± 3.0	<0.001
VE/VCO_2_ (slope, mean ± SD)	32.9 ± 4.7	36.1 ± 8.0	40.5 ± 9.3	<0.001
NT-proBNP (ng/L, mean ± SD)	1498.7 ± 1144.9	2275.2 ± 1910.3	3630.7 ± 2618.7	<0.001
Hemoglobin (g/dL, mean ± SD)	13.6 ± 1.7	13.4 ± 1.5	12.3 ± 1.7	0.003
Sodium (mEq/L, mean ± SD)	139.3 ± 2.8	138.7 ± 3.7	137.3 ± 3.7	0.047
eGFR (mL/min/1.73 m^2^, mean ± SD)	82.8 ± 23.8	65.3 ± 20.2	49.4 ± 18.3	<0.001
MECKI (mean ± SD)	8.6 ± 8.5	17.9 ± 11.1	25.5 ± 12.1	<0.001
MitraClip (%)	8.7	6.7	5.9	0.666
ICD (%)	73.9	83.3	90.2	0.073
CRT (%)	39.1	50.0	29.4	0.257
Events (%)	0.0	16.7	51.0	<0.001
Sacubitril/valsartan (%)	78.3	76.7	76.5	0.876
Furosemide (%)	65.2	93.3	94.1	0.002
Amiodarone (%)	13.0	40.0	47.1	0.019
Metolazone (%)	0.0	3.3	7.8	0.312
Ivabradine (%)	4.3	6.7	5.9	0.937
SGLT2 inhibitors (%)	69.6	50.0	47.1	0.096
MRAs (%)	82.6	75.9	74.0	0.720
Digoxin (%)	8.7	10.0	11.8	0.917
Beta-blockers (%)	95.7	86.7	90.2	0.588

Frailty categories were defined according to the frailty phenotype as robust (0 criteria), pre-frail (1–2 criteria), and frail (≥ 3 criteria). *p*-values refer to overall ANOVA group comparisons. NYHA = New York Heart Association, LVEF = left ventricular ejection fraction, TAPSE = Tricuspid Annular Plane Systolic Excursion, PASP = Pulmonary Arterial Systolic Pressure, IVCd = Inferior Vena Cava diameter, VO_2_max = maximal oxygen consumption, VE/VCO_2_ = minute ventilation/carbon dioxide production, NT-proBNP = N-terminal fragment of pro brain natriuretic peptide, eGFR = estimated Glomerular Filtration Rate, MECKI = Metabolic Exercise test data combined with Cardiac And Kidney Indexes, ICD = Implantable Cardioverter Defibrillator, CRT = Cardiac Resynchronization Therapy, Events = Death, urgent heart transplant, and left ventricular assisting device implantation, SGLT2 = Sodium-Glucose Transport Protein 2, MRAs = Mineralocorticoid receptor antagonist. CRT and ICD categories are not mutually exclusive.

**Table 3:** Association Between Physical Frailty and the Individual Components of the MECKI Score.

	Spearman Rank Correlations	
**Variables**	**ρ**	***p***
Hemoglobin	−0.351	<0.001
Sodium	−0.217	0.027
VO_2_max (% predicted)	−0.380	<0.001
VE/VCO_2_	0.463	<0.001
LVEF	−0.313	0.001
eGRF	−0.603	<0.001

ρ = Spearman’s rank correlation coefficient, VO_2_max = maximal oxygen consumption, VE/VCO_2_ = minute ventilation/carbon dioxide production, LVEF= Left Ventricular Ejection Fraction, eGFR = estimated Glomerular Filtration Rate.

**Table 4:** Cox Regression Analysis for Mortality and Urgent Transplantation or LVAD Implantation.

Variables	HR	95% CI	*p*
Age	0.917	0.873–0.964	0.001
Sex (Male)	0.664	0.258–1.707	0.395
NT-proBNP (ln)	1.025	1.010–1.041	0.001
Physical Frailty	1.843	1.249–2.721	0.002
MECKI	1.047	1.009–1.086	0.014

HR = hazard ratio; CI = confidence interval, NT-proBNP = N-terminal fragment of pro brain natriuretic, MECKI = Metabolic Exercise test data combined with Cardiac and Kidney Indexes.

## Data Availability

The data presented in this study are available on request from the corresponding author due to privacy and ethical restrictions.

## Supplementary Materials

The following supporting information can be downloaded at: https://www.mdpi.com/article/10.3390/jcm15020513/s1, Table S1: Baseline characteristics of patients excluded due to inability to perform CPET; Table S2: Bootstrap validation of the multivariable Cox regression model; Table S3: Multivariable Cox regression analysis for all-cause mortality (sensitivity analysis).

## Abbreviations

The following abbreviations are used in this manuscript:

HF	Heart Failure
PF	Physical Frailty
MF	Multidimensional Frailty
AHF	Advanced Heart Failure
HT	Heart Transplantation
LVAD	Left Ventricular Assist Device
MECKI	Metabolic Exercise Cardiac Kidney Index
VO_2_ max	Peak Oxygen Consumption
VE/VCO_2_	Minute Ventilation-Carbon Dioxide Production slope
CPET	CardioPulmonary Exercise Test
NT-pro-BNP	N-Terminal pro-B-type Natriuretic Peptide
eGFR	estimated Glomerular Filtration Rate
ROC	Receiver Operating Characteristic
LVEF	Left Ventricular Ejection Fraction
NYHA	New York Heart Association
TAPSE	Tricuspid Annular Plane Excursion
PASP	Pulmonary Arterial Systolic Pressure
