# Supplementary material for: Physical Frailty Versus the MECKI Score in Risk Stratification of Patients with Advanced Heart Failure: Simpler Measure, Similar Insights?
Source: J Clin Med. 2026 Jan 8;15(2):513. doi: 10.3390/jcm15020513 (PMC12842493; doi:10.3390/jcm15020513)
Supplement: Supplementary file 1 [file jcm-15-00513-s001.zip › jcm-4016867-supplementary.pdf]

Supplementary Table S1. Baseline characteristics of the study population compared with patients excluded from the analysis because of inability to perform cardiopulmonary exercise testing.

	**Study population (# 104)**	**Excluded population (# 20)**	**p**
Age (years, mean±SD)	58.4±12.0	68.5±8.2	<0.001
Male (%)	84.6	65.0	0.046
NYHA class (mean±SD)	2.9±0.6	3.5±0.5	<0.001
LVEF (%, mean±SD)	26.5±6.6	28.1±4.8	0.299
TAPSE (mm, mean±SD)	16.9±4.0	16.0±2.9	0.927
PASP (mmHg, mean±SD)	43.8±14.6	44.1±10.1	0.351
IVCd (mm, mean±SD)	20.0±4.8	21.5±2.3	0.181
NT-proBNP (ng/L, mean±SD)	2757.1±2337.5	2804.0±1602.4	0.873
Hemoglobin (g/dl, mean±SD)	12.9±1.7	10.4±1.4	<0.001
Sodium (mEq/L, mean±SD)	138.1±3.6	134.7±4.1	<0.001
eGFR (mL/min/1.73 m^2, mean±SD)	61.4±24.0	38.4±12.5	<0.001
Frailty	2.2±1.6	3.6±1.4	<0.001
MitraClip (%)	6.7	5.0	0.620
ICD (%)	84.6	70.0	0.089
CRT (%)	37.5	25.0	0.285
Sacubitril/valsartan (%)	76.9	45.0	0.004
Furosemide (%)	87.5	85.0	0.760
Amiodarone (%)	37.5	65.0	0.018
Metolazone (%)	4.8	10.0	0.357
Ivabradine (%)	5.8	10.0	0.481
SGLT2 inhibitors (%)	52.9	50.0	0.813
MRAs (%)	76.5	40.0	0.002
Digoxin (%)	10.6	15.0	0.400
Beta-blockers (%)	90.4	75.0	0.53

NYHA=New York Heart Association, LVEF=left ventricular ejection fraction, TAPSE=Tricuspid Annular Plane Systolic Excursion, PASP=Pulmonary Arterial Systolic Pressure, IVCd=Inferior Vena Cava diameter, NT-proBNP=N-terminal fragment of pro brain natriuretic peptide, eGFR=estimated Glomerular Filtration Rate, ICD=Implantable Cardioverter Defibrillator, CRT=Cardiac Resynchronization Therapy, SGLT2=Sodium-Glucose Transport Protein 2, MRAs=Mineralocorticoid receptor antagonist.

Supplementary Table S2. Bootstrap validation of the multivariable Cox regression model.

Variable	B (original)	SE (original)	p-value (original)	B (bootstrap)	95% CI (bootstrap)	p-value (bootstrap)
Age	−0.086	0.025	<0.001	−0.086	−0.151 to −0.034	0.003
Sex (Male)	−0.410	0.482	0.395	−0.410	−1.652 to 1.162	0.445
NT-proBNP (ln)	0.025	0.008	0.001	0.025	0.013 to 0.049	0.003
Physical Frailty	0.612	0.199	0.002	0.612	0.246 to 1.134	0.002
MECKI	0.046	0.019	0.014	0.046	0.009 to 0.096	0.015

Bootstrap results are based on 1000 resampled datasets.

B=regression coefficient, SE=standard error, CI=confidence interval, NT-proBNP=N-terminal fragment of pro brain natriuretic peptide, MECKI=Metabolic Exercise Cardiac Kidney Index.

Supplementary Table S3. Multivariable Cox regression analysis for mortality (sensitivity analysis).

Variable	HR	95% CI	p-value
Age	0.902	0.848–0.951	0.001
Sex (Male)	0.421	0.135–1.326	0.137
NT-proBNP (ln)	1.038	1.011–1.056	0.002
Physical Frailty	2.077	1.261–3.416	0.004
MECKI	1.072	1.024–1.126	0.003

Analyses were restricted to mortality, censoring patients at the time of urgent transplantation or LVAD implantation.

HR=hazard ratio; CI=confidence interval, NT-proBNP=N-terminal fragment of pro brain natriuretic peptide, MECKI=Metabolic Exercise Cardiac Kidney Index.
